# Rhizobial inoculation promotes glycyrrhizic acid production through jasmonic acid signaling in *Glycyrrhiza uralensis*

**DOI:** 10.1007/s11418-026-02072-z

**Published:** 2026-09-15

**Authors:** Shion Yamamoto, Aya Shimomura, Satoshi Watanabe, Nazmul Hasan, Akihiro Suzuki

**Affiliations:** 1 The United Graduate School of Agricultural Sciences, Kagoshima University, 1-21-24 Korimoto, Kagoshima, 890-0065 Japan; 2 Faculty of Agriculture, Saga University, 1 Honjo-machi, Saga, 840-8502 Japan

**Keywords:** *Glycyrrhiza*, Glycyrrhizic acid, Jasmonic acid, Rhizobium, Symbiosis

## Abstract

We previously found that inoculation with rhizobia tends to increase both biomass production and glycyrrhizic acid (GL) production in the medicinal plants *Glycyrrhiza uralensis* and *G. glabra*. In this study, we investigated the mechanism by which rhizobial inoculation promotes GL production. Transcriptome analysis of plants grown for 3, 6, 9, and 13 weeks after rhizobial inoculation revealed significant enrichment of GO terms related to root tissue differentiation and reorganization in inoculated plants. In addition, jasmonic acid (JA)-mediated signaling pathway, fatty acid biosynthetic process, and isoprenoid biosynthetic process were specifically enriched in inoculated plants, and these terms included *Jasmonate ZIM-domain protein* (*JAZ*)*-like*, *MYC-related transcription factor 2* (*MYC2*)*-like*, *Allene oxide cyclase* (*AOC*)*-like*, *Allene oxide synthase* (*AOS*)*-like*, and *Squalene synthase 2* (*SQS2*)*-like* genes. Time-course analysis of the expression patterns of these genes showed that *JAZ-like* genes were upregulated at the early growth stages in inoculated plants, whereas at 13 weeks after inoculation, expression of *JAZ-like* genes decreased while *MYC2b* and *CYP88D6* expression increased. These results suggest that *CYP88D6* expression may be regulated by *MYC2*. These results suggest that activation of JA biosynthesis and JA signaling is involved in the promotion of GL production by rhizobial inoculation, thereby supporting our previously proposed hypothesis at the transcriptome level. Further studies, including JA quantification, elucidation of transcriptional regulatory mechanisms, and functional analyses, will be necessary.

**Graphical abstract:**

**Figure:**
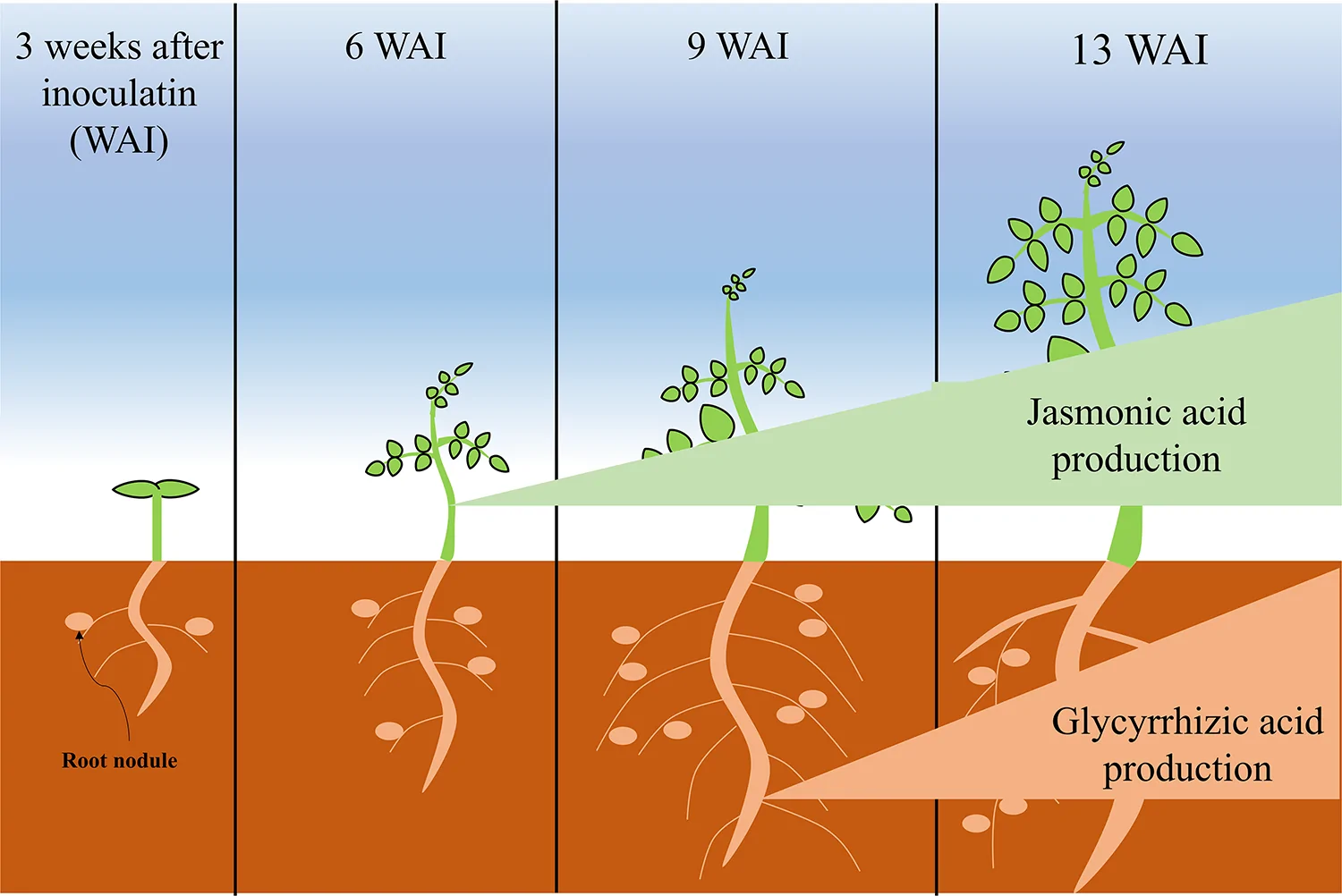

**Supplementary Information:**

The online version contains supplementary material available at https://doi.org/10.1007/s11418-026-02072-z.

## Introduction

*Glycyrrhiza* is a medicinal legume widely used in Japan as crude drug and is included in approximately 70% of Kampo medicines. In addition, global demand for licorice-derived products as natural sweeteners and cosmetic ingredients remains high, and the market continues to expand annually [1]. However, *Glycyrrhiza* does not grow naturally in Japan. *Glycyrrhiza uralensis* is native to Central Asia, northern China, and Mongolia, whereas *Glycyrrhiza glabra* is native to southern Europe and western China. Accordingly, the licorice used in Japan is supplied primarily through imports from China [2]. In recent years, overharvesting of wild populations has contributed to desertification and has made the stable supply of high-quality *Glycyrrhiza* increasingly difficult [3]. Therefore, the establishment of efficient domestic cultivation systems is essential for ensuring a stable supply of *Glycyrrhiza*.

*Glycyrrhiza* accumulates glycyrrhizic acid (GL), its principal bioactive constituent, in the roots and stolons. According to the Japanese Pharmacopoeia, licorice used as a crude drug must contain at least 2.0% GL on a root dry-weight basis (The Japanese Pharmacopoeia [4]). However, conventional cultivation requires at least three years to meet this standard, making reduction of the cultivation period a major challenge [5].

GL is a specialized metabolite. In higher plants, the production of specialized metabolites is generally closely associated with stress responses [6–8], and enhanced metabolite accumulation is often accompanied by reduced biomass production. A similar trade-off may therefore exist between GL accumulation and biomass production in licorice, and balancing these two traits is a key challenge in the development of efficient cultivation systems.

One potential strategy is the introduction of rhizobial symbiosis into *Glycyrrhiza* cultivation. Through symbiosis with rhizobia, *Glycyrrhiza* can obtain atmospheric nitrogen. Rhizobial symbiosis promotes host plant growth; however, rhizobial infection also represents a biotic stimulus and may affect specialized metabolism [9]. Thus, the establishment of root nodule symbiosis in *Glycyrrhiza* may enable simultaneous enhancement of biomass production and specialized metabolite accumulation. However, because nodule formation is rare in cultivated *Glycyrrhiza*, the effects of rhizobial symbiosis on GL production have not been fully investigated. We therefore isolated and identified *Mesorhizobium* sp. J8, a rhizobial strain capable of forming an efficient symbiotic association with *Glycyrrhiza*. Inoculation with J8 has been reported to enhance GL production in 90-day-old *G. uralensis* [10] and to promote both GL production and biomass accumulation in 150-day-old *G. glabra* [11].

GL is a triterpenoid saponin. Jasmonic acid (JA), a plant hormone, is known to promote the biosynthesis of terpenoid compounds [12]. In soybean, a leguminous species like *Glycyrrhiza*, rhizobial inoculation has been reported to enhance JA biosynthesis [13]. These findings suggest that JA may be involved in the rhizobium-induced promotion of GL production in *Glycyrrhiza*. Previous studies have shown that rhizobial inoculation significantly increases the expression of *Jasmonate Resistant 1* (*JAR1*) and *MYC-related transcription factor 2* (*MYC2*), both of which are involved in JA signaling [11]. In addition, methyl jasmonate (MeJA) treatment significantly upregulates *CYP72A154* and *CSyGT*, genes involved in GL biosynthesis. Together, these findings suggest that JA signaling contributes to the enhancement of GL production by rhizobial inoculation [11]. However, the underlying mechanism remains to be elucidated.

Because *Glycyrrhiza* is a non-model plant, genomic resources have historically been limited. However, recent advances in next-generation sequencing have enabled the publication of high-quality whole-genome sequences for *G. uralensis* and *G. glabra* [14–16], thereby facilitating genome-based transcriptome analyses.

In this study, we aimed to elucidate the mechanisms underlying the rhizobium-induced increase in GL production by performing transcriptome analysis using previously reported whole-genome datasets, with the goal of comprehensively characterizing the physiological responses of *Glycyrrhiza* to rhizobial inoculation.

## Material and methods

### Plant materials and growth conditions

*Glycyrrhiza uralensis* was used. For cultivation, vinyl pots (diameter 11 cm, height 24 cm) filled with vermiculite were used. Seedlings of *G. uralensis* were prepared using method described by Kusaba et al. [10]. The seeds were sown on water-agar medium and incubated for 3 days to promote root emergence. Seedlings at a similar developmental stage were selected and transplanted into vinyl pots. After transplantation, the plants were grown for 3, 6, or 9 weeks, or for up to 13 weeks. During the cultivation period, Broughton and Dilworth (B&D) liquid medium [17] containing 1 mM KNO₃ was supplied once per week. Plants were grown under a 16 h light/8 h dark photoperiod at 25 °C.

### Preparation and inoculation methods for rhizobia

The rhizobial strain used in this study was the same as that described by Kusaba et al. [10]. Bacterial culture, preparation, and inoculation were performed according to the methods reported by Kusaba et al. [10]. Rhizobial inoculation was conducted at the time of seedling transplantation.

### Measurement of GL content

GL content was determined by HPLC using a Primaide HPLC system (Hitachi High-Tech Corporation, Tokyo, Japan) equipped with an InertSustain C18 column (particle size, 5 μm; GL Sciences Inc., Tokyo, Japan). The mobile phase consisted of 0.1% (v/v) formic acid in water (solvent A) and acetonitrile (solvent B). Separation was performed using the following stepwise gradient program: 60% A and 40% B for 0–10 min, 50% A and 50% B for 10–30 min, 20% A and 80% B for 30–35 min, and 50% A and 50% B for 35–36 min. The mobile-phase composition was then returned to the initial conditions of 60% A and 40% B, and the column was re-equilibrated for 4 min. The column temperature was maintained at 40 °C, the flow rate was 0.6 ml/min, and detection was performed at 240 nm. Samples used for HPLC were prepared as follows: a 0.25 g of *G. uralensis* root powder was added to 35 ml of 50% (v/v) ethanol and shaken for 15 min. After shaking, the samples were centrifuged (6,000 rpm, 3 min) and the supernatant was transferred to a new tube. The remaining pellet was re-extracted with 12.5 ml of 50% (v/v) ethanol by shaking for 15 min, followed by centrifugation (6,000 rpm, 3 min). The second supernatant was combined with the first supernatant, and the extract was adjusted to a final volume of 50 ml with 50% (v/v) ethanol. The extract was then filtered through a membrane filter prior to HPLC analysis.

Glycyrrhizic Acid Standard for HPLC (FUJIFILM Wako Pure Chemical Corporation, Osaka, Japan) was used as an external standard. A 0.025% (w/v) standard solution was prepared in 50% (v/v) ethanol. The GL peak in each sample was identified by comparing its retention time with that of the standard. GL content was determined using a single-point external standard method by comparing the peak area of the sample with that of the standard solution.

### RNA-sequencing

Tap roots were collected at each growth stage, whereas lateral roots were excluded from the samples. The collected tap roots were immediately frozen in liquid nitrogen and stored at − 80 °C until use. The samples were homogenized using a Multi-Bead Shocker (Yasui Kiki, Japan). Total RNA was extracted using the RNeasy Plant Mini Kit (Qiagen, Basel, Switzerland), followed by DNase I treatment with the RNase-Free DNase Set (Qiagen, Basel, Switzerland). The extracted total RNA was subjected to RNA-seq analysis using the whole-genome sequence and annotation data of *G. uralensis* reported by Amit et al. (2022) [15]. Library preparation and sequencing were outsourced to BGI Japan Co., Ltd., and libraries were prepared using an mRNA enrichment method.

### Principal component analysis (PCA)

Principal component analysis (PCA) was performed using normalized RNA-seq expression data. Gene expression values were log2-transformed, and the variance of each gene was calculated. The 1,000 genes with the highest variance were selected for PCA, which was conducted using the prcomp function in R version 4.2.2. Samples were visualized based on the first and second principal components.

### Analysis of differentially expressed genes (DEGs)

Raw reads obtained from BGI Japan were processed to generate clean reads by removing adapter sequences, low-quality reads, and reads containing undetermined bases. Differentially expressed gene (DEG) analysis was then performed using the clean reads with the online RNA-seq analysis platform RaNA-seq [18].

### Analysis of gene ontology (GO) enrichment

Gene annotation was performed using the *G. uralensis* annotation dataset available in the UniProt database. Gene Ontology (GO) enrichment analysis was subsequently conducted based on the resulting annotation data.

### Comparative analysis using public *Lotus japonicus* RNA-seq data

Processed RNA-seq count data for *Lotus japonicus* MG20 were obtained from the NCBI Gene Expression Omnibus under accession number GSE129778 and independently reanalyzed in the present study. The dataset contained count data for uninoculated roots and nodules at 5, 7, and 10 days after inoculation with *Mesorhizobium loti*. Expression changes between nodule developmental stages were calculated using normalized count values, and genes with increased expression were subjected to GO enrichment analysis using Lotus Base annotation.

### Heatmap analysis

A heatmap was generated in R using count data obtained from RaNA-seq. For each treatment group, expression levels at 6, 9, and 13 WAI were normalized to the corresponding 3 WAI sample and log2-transformed after adding a pseudocount of 1.

### Phylogenetic analysis

Amino acid sequences were aligned using MAFFT version 7.526 [19], and phylogenetic trees were constructed from the resulting alignments using IQ-TREE version 3.1.1 [20]. Domain analyses were performed using the predicted amino acid sequences of candidate genes obtained from the genome annotation data of *G. uralensis*. Conserved domains were identified using NCBI Conserved Domain Search (CD-Search) [21]. Specifically, the TIFY domain and Jas motif were examined in JAZ candidates, the bHLH-MYC_N domain in MYC2 candidates, the allene oxide cyclase domain in AOC candidates, the CYP74 domain and heme-binding motif in AOS candidates, and the SQS/PSY domain in SQS2 candidates. Amino acid sequence alignments were visualized using Jalview [22], and residues were colored according to the Clustal color scheme.

### qPCR

Gene expression analysis was performed using the One Step TB Green PrimeScript PLUS RT-PCR Kit (Takara, Shiga, Japan), and quantitative PCR (qPCR) was conducted on an Applied Biosystems StepOne Real-Time PCR System (Thermo Fisher Scientific, MA, USA). The gene-specific primer sequences used in this study are listed in Table 1. Relative expression levels were calculated using the 2 − ΔΔCt method [23]. Expression levels of target genes were normalized to 18 S rRNA, and the 3 WAI sample was used as the calibrator. The relative expression level at 3 WAI was set to 1.

**Table 1:** Primer sequences used in this study

No	Target gene	Forward primer sequence (5'−3')	Reverse primer sequence (5'−3')
1	GuJAZ10/11/12-like	ATGTTGGTGGAAGGAGCAGTG	GTTGGCATTGGCCAGTTAGC
2	GuJAZ1-like	CATCCCCACCACTGGCACC	AGCAATTCTATCCTTTCTCTTCTCC
3	GuJAZ1/2-like1	CTCCCTTAGAAACACCTCGAC	GTCCTGTGGCAATGACCCTTC
4	GuJAZ1/2-like2	CATTGGCCAGCAAAGGAACC	AGCAATTGGTAGGTCACAAGC
5	GuJAZ1/2-like3	CGTCCCTACCACTGCCAAC	GCCCTGGCAGCAATTCTATCCTTTC
6	GuMYC2a	AGGTAACCGACACCGAGTGG	ATTTGTCAGCCCCGGAGACC
7	GuMYC2b	GCAGTTAATTCCGGCCACCG	CGTCGTTGTTGCTTCCC
8	GuAOCa	ACAAATTGTACACTGGGGACCTG	AGCCAAGTGACCGTAGTCTC
9	GuAOCb	CCGTGGTAGCCCTGCATACCTG	CTGAACGGCCAAGTGACCGTAGTCC
10	GuAOS1	GGTCTTCCGAGCAAACATGC	AGTTACGTCGAAGAGGATTGG
11	GuAOS3	CGCTGCATGAGAGTTCTCC	ATTCAGAGGCCACCGTTTCC
12	GuSQSa	TGGTTCTTCGAGCCCTTGAC	TGCCACAGGAAAAGTGCCAG
13	GuSQSb	TGGCACTTTTCATGTGGCACG	ACCCATTCTTTTGGTAATG
14	GuCYP88D6	GTTTCCTTTAAAGCCATTGTCCACGTC	ATCTTTTCTTTGGCTCCCTTCTTGTG
15	GuCYP72A154	CTTCCACACCAGGGGCTATCTG	CTTGCCCTTGAGCTCTGAGATG
16	18S rRNA	CTCAACACGGGGAAACTTAC	AGACAAATCGCTCCACCAAC

Primer sequences are shown in the 5' and 3' direction.Amplicon sizes are not included because they were not provided in the source table

## Results

### Effects of rhizobial inoculation on plant growth, nodulation, and GL content at 13 WAI

**Fig. 1:**
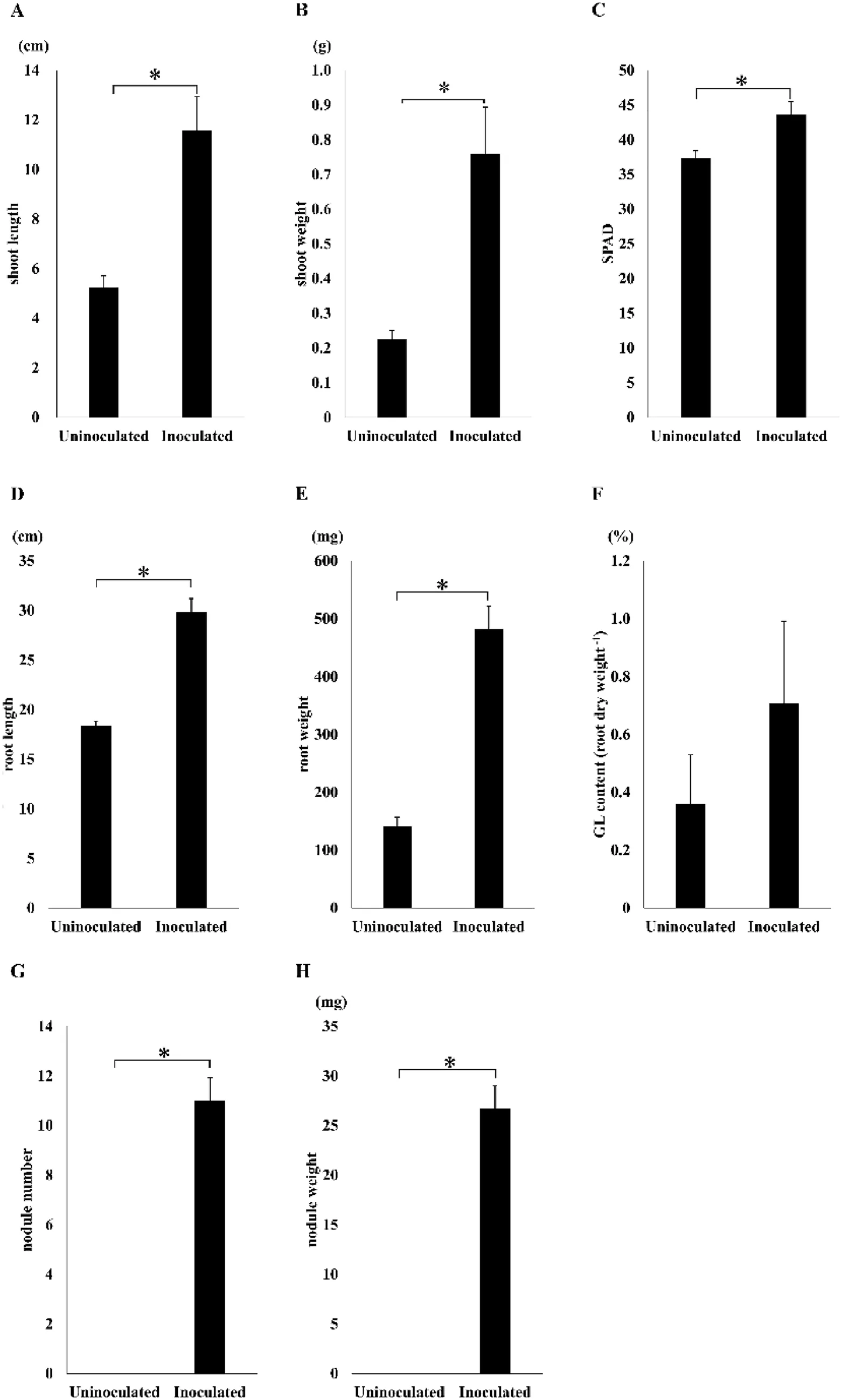
Effects of rhizobial inoculation on plant growth and GL content in *Glycyrrhiza uralensis* at 13 WAI. Shoot length (**A**), shoot weight (**B**), SPAD value (**C**), root length (**D**), root weight (**E**), and GL content (**F**) were measured in inoculated and uninoculated plants. Error bars indicate standard errors. * indicates significant differences between inoculated and uninoculated plants (Student’s *t*-test: *p < 0.05) (*n* = 3)

At 13 weeks after inoculation (13 WAI), rhizobial inoculation significantly increased shoot length, shoot weight, SPAD value, root length, and root weight compared with uninoculated plants (Fig. 1A–E). GL content tended to be higher in inoculated plants than in uninoculated plants (Fig. 1F). Nodules were observed only in inoculated plants, and nodule number and nodule weight were significantly higher in inoculated plants than in uninoculated plants (Fig. 1G, H). These results indicate that rhizobial inoculation induced nodulation, promoted plant growth, and tended to increase GL accumulation under the conditions used in this study.

### Analysis of differentially expressed genes across developmental stages

Based on the observed growth-promoting effects of rhizobial inoculation at 13 WAI, together with our previous findings suggesting the involvement of jasmonic acid (JA) in rhizobium-induced GL production [11], we performed transcriptome analysis to investigate the molecular responses underlying these effects.

**Fig. 2:**
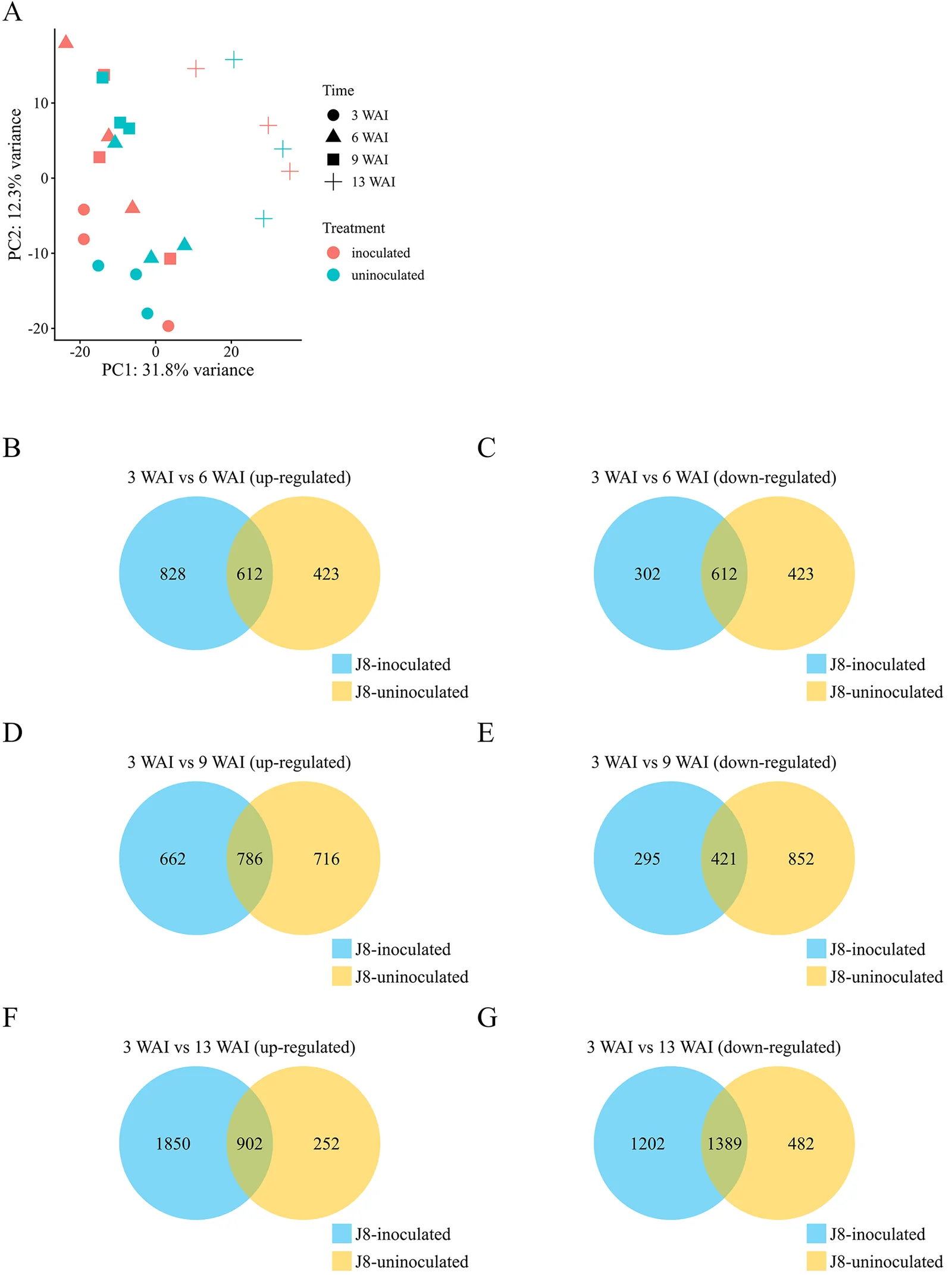
Principal component analysis of RNA-seq data and analysis of differentially expressed genes **A** Principal component analysis of samples from inoculated and uninoculated plants at each growth stage. **B**–**G** Venn diagrams showing the numbers of genes that were significantly differentially expressed between 3 weeks after inoculation (WAI) and 6, 9, or 13 WAI in inoculated and uninoculated plants (*n* = 3). Blue circles indicate genes that were differentially expressed in inoculated plants, whereas yellow circles indicate genes that were differentially expressed in uninoculated plants. The compared growth stages are indicated below each diagram. The numbers of upregulated genes are shown for the comparisons of 3 vs. 6 WAI **B**, 3 vs. 9 WAI **D**, and 3 vs. 13 WAI **F**, and the numbers of downregulated genes are shown for the comparisons of 3 vs. 6 WAI **C**, 3 vs. 9 WAI **E**, and 3 vs. 13 WAI **G**

RNA-seq was performed using inoculated and uninoculated plants grown for 3, 6, 9, and 13 weeks. Principal component analysis (PCA) based on the 1,000 most variable genes showed that PC1 and PC2 accounted for 31.8% and 12.3% of the total variance, respectively (Fig. 2A). Samples tended to separate according to growth stage along PC1 and according to inoculation status along PC2. Based on these results, differentially expressed genes were identified in inoculated and uninoculated plants by using 3 WAI as the reference time point and comparing it with 6, 9, and 13 WAI. In the comparison between 3 and 6 WAI, 1,130 genes exhibited inoculation-specific differential expression, including 828 upregulated and 302 downregulated genes, whereas 846 genes exhibited uninoculated-specific differential expression, including 423 upregulated and 423 downregulated genes (Fig. 2B, C). In the comparison between 3 and 9 WAI, 957 genes showed inoculation-specific differential expression, of which 662 were upregulated and 295 were downregulated, whereas 1,568 genes showed uninoculated-specific differential expression, including 716 upregulated and 852 downregulated genes (Fig. 2D, E). In the comparison between 3 and 13 WAI, 3,052 genes exhibited inoculation-specific differential expression, including 1,850 upregulated and 1,202 downregulated genes, whereas 734 genes exhibited uninoculated-specific differential expression, including 252 upregulated and 482 downregulated genes (Fig. 2F, G).

### Gene ontology (GO) enrichment analysis

**Fig. 3:**
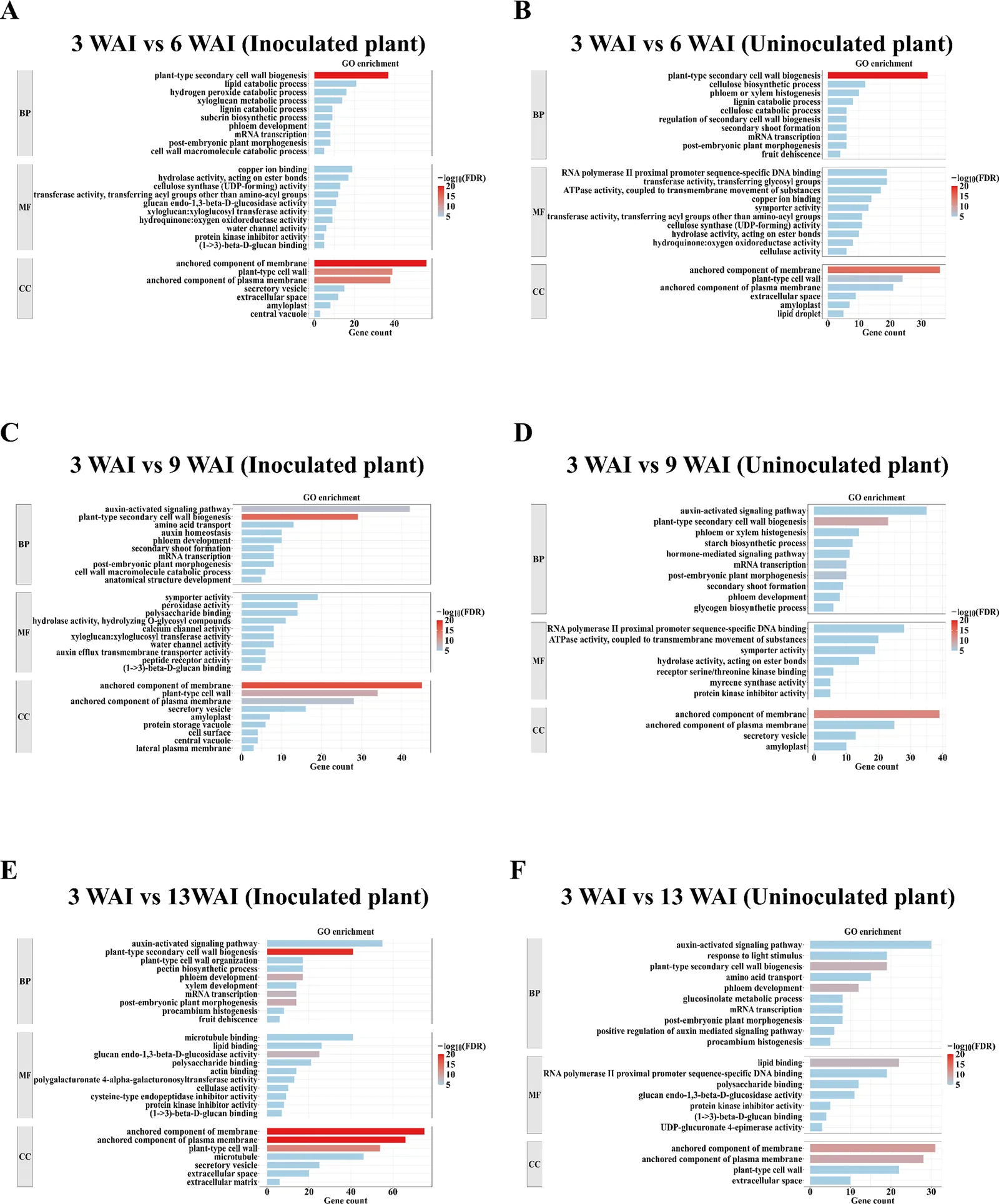
Gene Ontology enrichment analysis of genes upregulated at 6, 9, and 13 weeks after inoculation (WAI) relative to 3 WAI in inoculated and uninoculated plants Representative enriched terms for biological process (BP), molecular function (MF), and cellular component (CC) are shown for inoculated plants at 3 vs. 6 WAI (**A**), uninoculated plants at 3 vs. 6 WAI (**B)**, inoculated plants at 3 vs. 9 WAI (**C**), uninoculated plants at 3 vs. 9 WAI (**D**), inoculated plants at 3 vs. 13 WAI (**E**), and uninoculated plants at 3 vs. 13 WAI (**F**). Bar length indicates the number of genes, and bar color indicates enrichment significance [− log10(FDR)]

Among the identified DEGs, we focused on genes that were upregulated in both inoculated and uninoculated plants and subjected them to Gene Ontology (GO) enrichment analysis. At all examined time points relative to 3 WAI—namely, 6, 9, and 13 WAI—GO terms such as “plant-type secondary cell wall biogenesis,” “phloem development,” and “anchored component of membrane”/“anchored component of plasma membrane” were enriched in both inoculated and uninoculated plants. These results indicate that root development, vascular tissue formation, cell wall biosynthesis, and membrane reorganization progressed after 3 WAI irrespective of inoculation treatment (Fig. 3). In the comparison between 3 and 6 WAI, inoculated plants were additionally characterized by enrichment of GO terms related to lipid catabolism, hydrogen peroxide catabolism, xyloglucan metabolism, lignin catabolism, suberin biosynthesis, water channel activity, and secretory vesicles/extracellular space, suggesting that rhizobial inoculation was accompanied by tissue remodeling involving cell wall modification, stress-related responses, and secretory activity (Fig. 3A, B). In the comparison between 3 and 9 WAI, terms related to auxin homeostasis, auxin efflux transporter activity, xyloglucan: xyloglucosyl transferase activity, and water channel activity were enriched in inoculated plants, suggesting that rhizobial inoculation promotes tissue remodeling involving auxin regulation, membrane transport, and cell wall reorganization. By contrast, terms such as “starch biosynthetic process” and “amyloplast” were enriched in uninoculated plants, suggesting the progression of more general maturation and storage metabolism (Fig. 3C, D). In the comparison between 3 and 13 WAI, GO terms related to plant-type cell wall organization, pectin biosynthetic process, microtubule binding, actin binding, and secretory vesicles were enriched in inoculated plants, further suggesting that rhizobial inoculation induces tissue remodeling associated with cell wall reorganization, cytoskeletal dynamics, and secretory transport (Fig. 3E, F). Collectively, these results indicate that, after 3 WAI, both inoculated and uninoculated licorice roots underwent transcriptomic changes associated with development and cell wall formation, whereas inoculated roots additionally exhibited extensive tissue remodeling accompanied by auxin-related regulation, cell wall reconstruction, cytoskeletal reorganization, and activation of the secretory system.

In the comparison between 3 and 6 WAI, several GO terms were specifically enriched in inoculated plants (Supplementary Table 1). Based on our previous finding that JA may be involved in the rhizobial inoculation-induced promotion of GL production [11], we focused on three inoculation-specific GO terms: “fatty acid biosynthetic process”, which may be related to precursor supply for JA biosynthesis; “jasmonic acid (JA)-mediated signaling pathway”, which is associated with JA responses; and “isoprenoid biosynthetic process”, which may be related to formation of the triterpenoid skeleton of GL. Genes associated with these terms included *Jasmonate ZIM-domain protein-like* genes (*JAZ-like*; *Glur_chr1.g095130*, *Glur_chr7.g008620*, *Glur_chr1.g071860*, *Glur_chr1.g094600*, and *Glur_chr6.g075300*) and *MYC2-like* genes (*Glur_chr1.g059400.1* and *Glur_chr6.g066530.1*), which are involved in JA signaling; *Allene oxide cyclase-like* genes (*AOC-like*; *Glur_chr2.g068350.1* and *Glur_chr2.g006050.1*) and *Allene oxide synthase-like* genes (*AOS-like*; *Glur_chr4.g002340.1* and *Glur_chr2.g060100.1*), which participate in JA biosynthesis; and *Squalene synthase 2-like* genes (*SQS2-like*; *Glur_chr5.g080790.1* and *Glur_chr3.g069060.1*), which are involved in the biosynthesis of squalene, a precursor of GL. These findings suggest that JA biosynthesis and JA signaling may contribute to the rhizobium-induced enhancement of GL production.

**Fig. 4:**
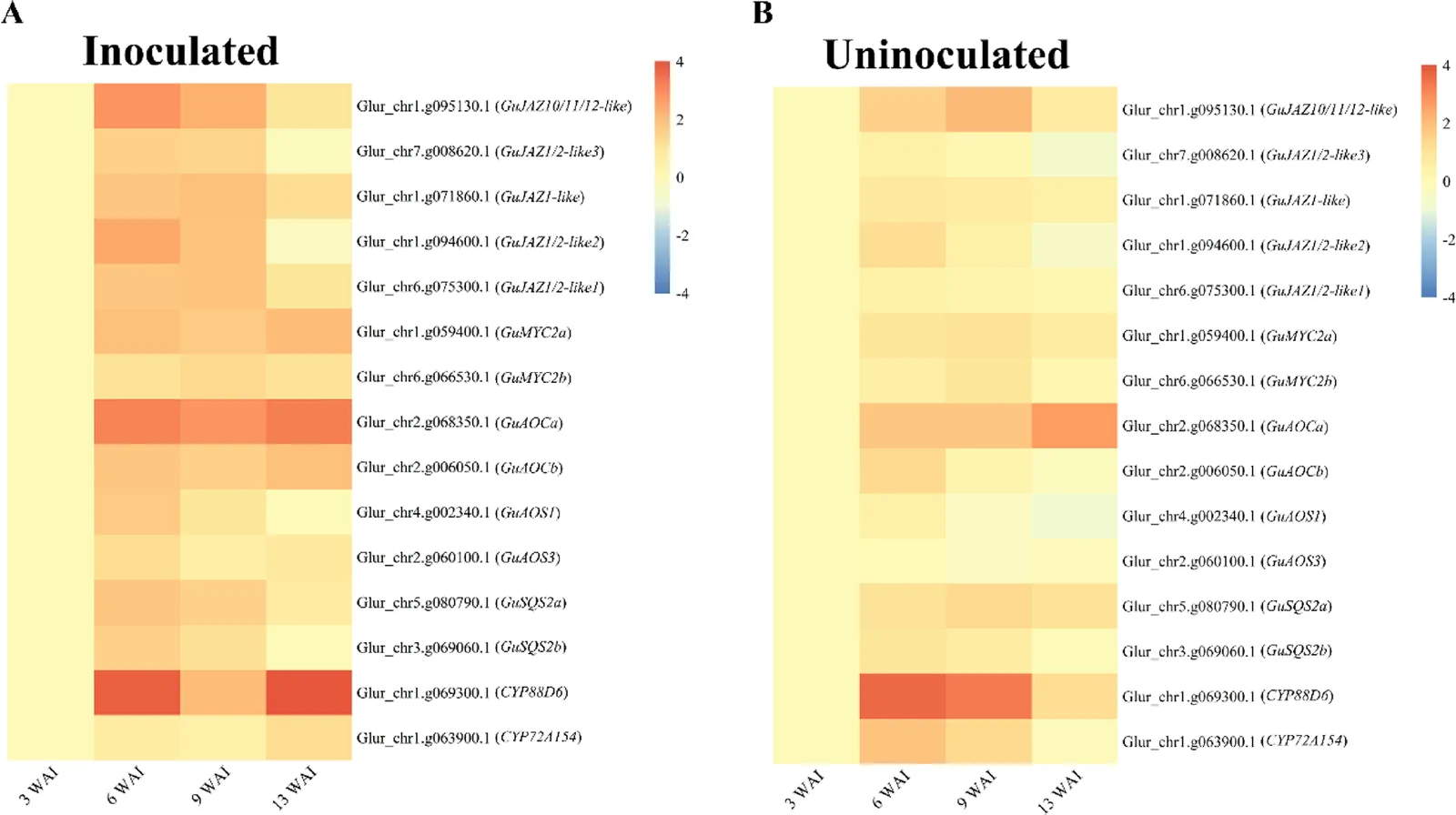
Expression patterns of JA-related genes, triterpenoid biosynthesis-related genes, and GL biosynthesis-related genes during plant growth in inoculated and uninoculated plants. Heatmaps show the relative expression levels of each gene across growth stages, with colors indicating expression levels from low to high. Inoculated plants are shown in (**A**), and uninoculated plants are shown in (**B**). The color bar indicates log2-transformed relative expression levels normalized to the corresponding 3 WAI sample within each treatment group

We next examined growth stage-dependent expression patterns of the aforementioned *JAZ-like*, *MYC2-like*, *AOC-like*, *AOS-like*, and *SQS2-like* genes, together with the GL biosynthesis-related genes *CYP88D6* (*Glur_chr1.g069300.1*) and *CYP72A154* (*Glur_chr1.g063900.1*), using RNA-seq data (Fig. 4). In inoculated plants, many of these genes showed a general increase in expression as development progressed (Fig. 4A). By contrast, in uninoculated plants, the expression of GL biosynthesis-related genes tended to decrease with advancing growth stage (Fig. 4B). Notably, the expression of *JAZ-like*, *MYC2-like*, *AOC-like*, and *AOS-like* genes was elevated at 6 and 9 WAI in inoculated plants. Taken together, these results suggest that rhizobial inoculation induces genes associated with JA biosynthesis and JA signaling.

### Phylogenetic analysis of candidate gene families

**Fig. 5:**
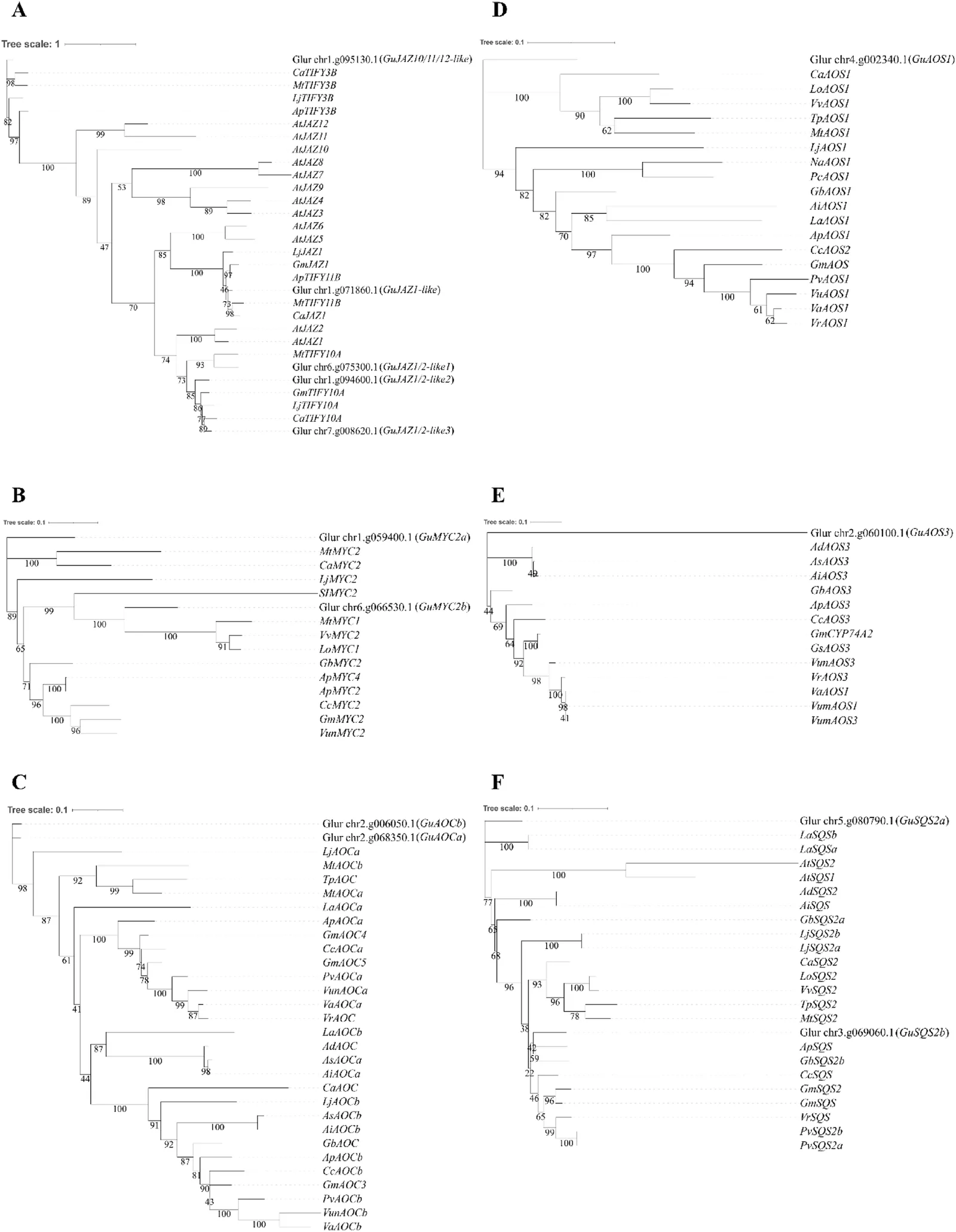
Phylogenetic analysis of JA-related and triterpenoid biosynthesis-related genes identified in *Glycyrrhiza uralensis*. Phylogenetic trees were constructed using the amino acid sequences of candidate genes identified in this study and known homologs reported in legume and non-legume plant species. Separate phylogenetic trees are shown for *JAZ-like* genes (**A**), *MYC2-like* genes (**B**), *AOC-like* genes (**C**), *AOS1-like* genes (**D**), *AOS3-like* genes (**E**), and *SQS2-like* genes (**F**). Candidate genes identified in *G. uralensis* are indicated by their respective gene IDs. The gene IDs and accession numbers of the sequences used for phylogenetic analysis are listed in Supplementary Table 2. Numbers at the nodes indicate bootstrap values, and scale bars represent the number of amino acid substitutions per site

GO enrichment analysis identified *JAZ-like*, *MYC2-like*, *AOC-like*, *AOS-like*, and *SQS2-like* genes whose expression was induced by rhizobial inoculation. To validate the annotation of these candidate genes, phylogenetic analyses were performed using their predicted amino acid sequences together with previously characterized proteins from legume and non-legume plant species (Fig. 5). All five *JAZ-like* genes clustered within the JAZ clade (Fig. 5A). Based on their phylogenetic relationships with known JAZ/TIFY proteins, these genes were tentatively designated *GuJAZ10/11/12-like* (*Glur_chr1.g095130.1*), *GuJAZ1-like* (*Glur_chr1.g071860.1*), *GuJAZ1/2-like1* (*Glur_chr6.g075300.1*), *GuJAZ1/2-like2* (*Glur_chr1.g094600.1*), and *GuJAZ1/2-like3* (*Glur_chr7.g008620.1*). Both *MYC2-like* genes clustered within the *MYC2* clade (Fig. 5B) and were tentatively designated *GuMYC2a* (*Glur_chr1.g059400.1*) and *GuMYC2b* (*Glur_chr6.g066530.1*) based on their relationships with known MYC family proteins. Similarly, both *AOC-like* genes clustered within the *AOC* clade (Fig. 5C) and were tentatively designated *GuAOCa* (*Glur_chr2.g068350.1*) and *GuAOCb* (*Glur_chr2.g006050.1*). Among the *AOS-like* genes, *Glur_chr4.g002340.1* clustered within the *AOS1* clade, whereas *Glur_chr2.g060100.1* clustered within the *AOS3* clade (Fig. 5D, E). These genes were therefore tentatively designated *GuAOS1* and *GuAOS3*, respectively. Both *SQS2-like* genes clustered within the *SQS2* clade (Fig. 5F) and were tentatively designated *GuSQS2a* (*Glur_chr5.g080790.1*) and *GuSQS2b* (*Glur_chr3.g069060.1*) based on their phylogenetic relationships with known SQS2 proteins.

Although all candidate genes showed close phylogenetic relationships with previously characterized genes, some were located near the periphery of their respective clades. We therefore further examined whether they retained the conserved domains characteristic of each gene family (Supplementary Fig. 1). The predicted amino acid sequences of the GuJAZ proteins contained both the TIFY domain and Jas motif, which are conserved features of JAZ proteins (Supplementary Fig. 1 A). The GuMYC2 proteins contained the bHLH-MYC_N domain characteristic of MYC proteins (Supplementary Fig. 1B), and the GuAOC proteins contained the allene oxide cyclase domain characteristic of AOC proteins (Supplementary Fig. 1 C). The GuAOS proteins contained the CYP74 domain characteristic of AOS proteins. However, the heme-binding motif was detected only in GuAOS1 and not in GuAOS3 (Supplementary Fig. 1D, E). The GuSQS2 proteins contained the SQS/PSY domain characteristic of SQS2 proteins (Supplementary Fig. 1 F). Taken together, these results support the assignment of most candidate genes to their corresponding gene families.

### Growth stage-dependent expression profiles of candidate genes

**Fig. 6:**
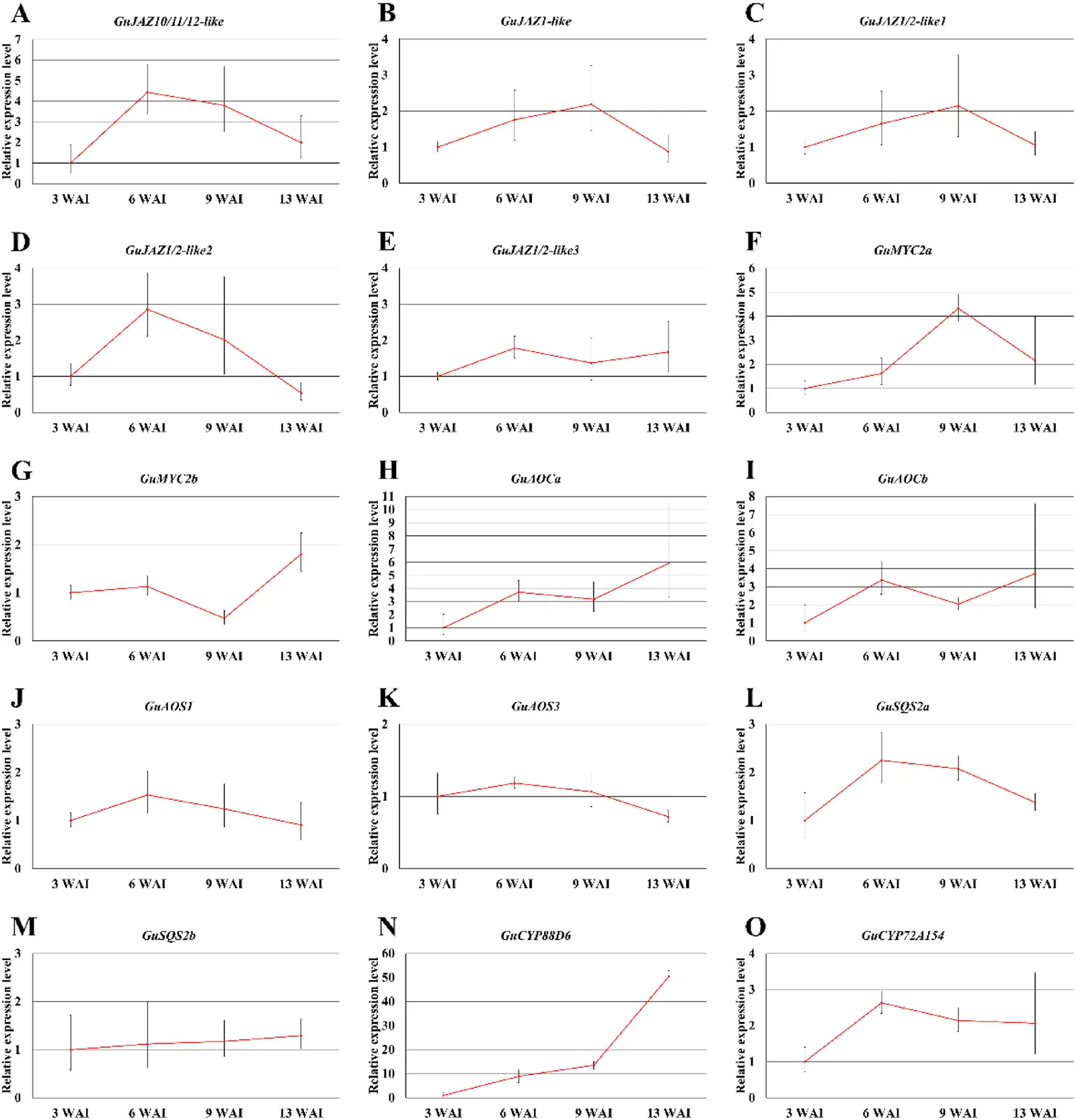
Relative expression changes in JA-related genes, triterpenoid biosynthesis-related genes, and GL biosynthesis-related genes during growth in inoculated and uninoculated plants. For each gene, expression levels at 3 weeks after inoculation (WAI) were set to 1, and relative expression levels at 6, 9, and 13 WAI are shown. *18 S rRNA* gene was used as the internal control. The panels show *GuJAZ* gene clusters (**A**–**E**), *GuMYC2* gene clusters (**F**, **G**), *GuAOC* gene clusters (**H**, **I**), *GuAOS1* and *GuAOS3* (**J**, **K**), *GuSQS2* gene clusters (**L**, **M**), *GuCYP88D6* (**N**), and *GuCYP72A154* (**O**). Values are means, and error bars indicate standard errors (*n* = 3)

Expression profiles of the *GuJAZ*, *GuMYC2*, *GuAOC*, *GuAOS1*, *GuAOS3*, and *GuSQS2* genes, together with those of the GL biosynthesis-related genes *CYP88D6* and *CYP72A154*, were analyzed across growth stages in inoculated plants, with expression levels at 3 WAI normalized to 1. The *GuJAZ10/11/12-like*, *GuJAZ1-like*, *and GuJAZ1/2-like1*,*2* showed similar expression patterns, whereas their expression tended to increase at 6 and 9 WAI. By contrast, *GuJAZ1/2-like3* showed no marked expression changes (Fig. 6A-E). The *GuMYC2a* gene showed that expression tended to increase at 6 and 9 WAI, while the *GuMYC2b* gene showed that expression tended to increase at 13 WAI (Fig. 6F, G). The *GuAOC* and *GuAOS* genes exhibited generally similar expression patterns (Fig. 6H-K). While *GuSQS2a* showed that expression tended to increase at 6 and 9 WAI, *GuSQS2b* exhibited a different pattern, with no marked changes in expression levels from 3 WAI to 13 WAI (Fig. 6L, M). *GuCYP88D6* showed increased expression at 13 WAI (Fig. 6N). *GuCYP72A154* also showed the highest expression at 6 WAI, and its expression level was higher at 13 WAI than at 3 WAI (Fig. 6O).

To compare the present results with our previous findings in *G. glabra*, the expression levels of *JAR1* and *CSyGT* were examined at 3 WAI. *CSyGT* showed a slight increase in inoculated plants, whereas *JAR1* was not upregulated by rhizobial inoculation (Supplementary Fig. 2).

These results suggest that increased expression of *GuCYP88D6* at 13 WAI coincided with increased expression of *GuMYC2b*, indicating a possible association between *GuCYP88D6* expression and JA signaling-related transcriptional regulation.

## Discussion

In this study, principal component analysis indicated that the physiological state of licorice was influenced by both developmental stage and rhizobial inoculation. However, some uninoculated samples were not clearly separated from inoculated samples. This may have resulted from partial activation of symbiotic responses due to rhizobial contamination in uninoculated plants, as well as from genetic heterogeneity among the *G. uralensis* individuals used in this study. Indeed, previous studies have reported that wild licorice populations contain numerous hybrid individuals of *G. uralensis* and *G. glabra* [24], suggesting that responsiveness to rhizobial inoculation may also vary among individuals. Future studies should therefore select individuals with high rhizobial responsiveness and further characterize their genetic backgrounds to enable a more precise evaluation of the effects of rhizobial inoculation on licorice physiology and GL production.

GO enrichment analysis suggested that the transcriptomic changes observed during plant growth largely reflected ongoing root development. With respect to inoculation-dependent differences, the comparison between 3 and 6 WAI revealed specific enrichment of the “JA-mediated signaling pathway,” “fatty acid biosynthetic process,” and “isoprenoid biosynthetic process” in inoculated plants. Genes associated with these terms included *JAZ-like*, *MYC2-like*, *AOC-like*, *AOS-like*, and *SQS2-like* genes. Moreover, time-course analysis of the RNA-seq data showed that the expression of *JAZ-like*, *MYC2-like*, *AOC-like*, and *AOS-like* genes was elevated at 6 and 9 WAI in inoculated plants. These findings support the possibility that rhizobial inoculation activates JA biosynthesis and JA signaling, thereby providing transcriptome-level evidence for our previous hypothesis that JA contributes to the rhizobium-induced enhancement of GL production [11]. In particular, the upregulation of *JAZ-like* genes at 6 and 9 WAI suggests that JA responses are activated at a relatively early stage following rhizobial inoculation. The inoculation-associated enrichment of terms related to auxin responses, cell wall organization, cytoskeletal dynamics, and secretory processes further suggests that rhizobial inoculation is accompanied by extensive root tissue remodeling. However, these transcriptional changes may reflect developmental programs associated with symbiotic organogenesis, and whether they directly contribute to enhanced GL accumulation remains to be determined.

In our previous study using *G. glabra*, rhizobial inoculation increased the expression of *JAR1* at 30 days after inoculation, and MeJA treatment induced the expression of GL biosynthesis-related genes, including *CSyGT* [11]. Therefore, in the present study, we further examined the expression of *JAR1* and *CSyGT* at 3 WAI in *G. uralensis*. At this stage, *CSyGT* showed a slight increase in inoculated plants, whereas *JAR1* was not upregulated by rhizobial inoculation. These results indicate that the expression responses of *JAR1* and *CSyGT* at 3 WAI in *G. uralensis* were not fully consistent with those observed in our previous study using *G. glabra*. However, this difference should be interpreted cautiously because the previous study used *G. glabra* plants prepared from cuttings, whereas the present study used seed-derived *G. uralensis* plants. Since seed-derived *G. uralensis* plants are not genetically fixed, genetic variation among individual plants may have affected their transcriptional responses to rhizobial inoculation. Therefore, future studies using genetically fixed or genetically uniform *G. uralensis* lines will be important for more precisely evaluating the effects of rhizobial inoculation on JA-related gene activation and GL biosynthesis-related gene expression.

Among the GL biosynthesis-related genes, *CYP88D6* exhibited a sustained increase in expression from 3 to 13 WAI in inoculated plants. In addition, although *JAZ-like* gene expression decreased at 13 WAI in inoculated plants, *GuMYC2b* expression increased at the same stage. These observations suggest that *CYP88D6* expression may be associated with transcriptional regulation related to JA signaling. By contrast, *CYP72A154* showed a slight increase in expression levels, suggesting that the regulation of downstream GL biosynthetic genes may not be uniform. This difference may reflect gene-specific regulatory mechanisms, distinct temporal responses to JA signaling, or differences between endogenous responses to rhizobial inoculation and responses induced by exogenous MeJA treatment. JAZ proteins have been reported to repress the expression and activity of MYC2 [25]. Because *JAZ* genes are themselves induced by JA and function as negative regulators of JA signaling, their increased expression at 6 and 9 WAI may reflect activation of a feedback regulatory loop rather than simply sustained enhancement of JA signaling. The subsequent decrease in *JAZ-like* expression together with the increase in *GuMYC2b* expression at 13 WAI may therefore indicate a temporal reconfiguration of JA signaling during later stages of the symbiotic response. This expression pattern suggests that JAZ-like genes and *GuMYC2b* may also participate in JA signaling in *G. uralensis*. However, the present study demonstrates only coordinated expression patterns and does not establish direct regulation of *CYP88D6* by GuMYC2b. Analysis of the region predicted to be the promoter, extending 2,000 bp upstream of the start codon of *GuCYP88D6*, revealed five MYC-related elements and two elements associated with MeJA response. Previous studies have shown that increased endogenous JA levels enhance GL production in *Glycyrrhiza* plants [26], supporting our hypothesis that rhizobial inoculation promotes GL biosynthesis through the induction of JA biosynthesis-related genes and activation of JA signaling. Further functional analyses, including promoter assays and transcriptional activation experiments, will be required to clarify the regulatory relationships among these factors.

Phylogenetic and conserved-domain analyses showed that many of the *JAZ-like*, *MYC2-like*, *AOC-like*, *AOS-like*, and *SQS2-like* candidate genes were closely related to their corresponding known gene families and retained the conserved domains characteristic of those families. These findings indicate that these genes are strong candidates for involvement in JA responses and triterpenoid biosynthesis in licorice. By contrast, the absence of a heme-binding motif in GuAOS3 suggests that this protein may have functional properties distinct from those of canonical AOS proteins. Further functional characterization of these candidate genes will therefore be required.

Although differences in growth stage and sample type should be taken into account, comparative GO enrichment analysis of *Lotus japonicus* MG20 [27, 28] and our RNA-seq data from *G. uralensis* following rhizobial inoculation revealed both shared and distinct transcriptional features. A common feature in both species was the enrichment of terms related to auxin responses and membrane transport, suggesting that auxin-mediated regulation and transport processes may be involved in root development and tissue reorganization. In contrast, whereas terms associated with metal ion transport and redox regulation were prominent in MG20, terms related to vascular development, cell wall remodeling, cytoskeletal changes, and activation of the secretory system were more characteristic of *G. uralensis*. Furthermore, in *G. uralensis*, in addition to JA (jasmonic acid)-related terms, induction of gene clusters associated with JA biosynthesis and JA signaling was also observed. Taken together, these findings suggest that, whereas relatively early environmental responses and metabolic regulation are more prominent in *L. japonicus*, tissue reorganization and metabolic control accompanied by JA responses may be more strongly developed in *G. uralensis*.

The present findings may also have implications for overcoming the apparent trade-off between biomass accumulation and specialized metabolite production in licorice. Rhizobial symbiosis has the potential to improve plant nutritional status while simultaneously activating signaling pathways associated with GL biosynthesis, which may provide a physiological basis for achieving both enhanced growth and increased metabolite accumulation. However, because carbon and nitrogen allocation were not directly examined in this study, the relationship between symbiotic nutrient acquisition and GL production remains to be clarified. Taken together, our results suggest that the rhizobium-induced enhancement of GL production involves activation of JA biosynthesis and JA signaling, while also indicating the possible involvement of developmental regulation, including auxin-related responses, cell wall reorganization, cytoskeletal dynamics, and activation of the secretory system. However, this study was based primarily on RNA-seq analysis and did not directly assess temporal changes in JA content. Moreover, the regulatory relationship between *MYC2* and *CYP88D6*, as well as the molecular functions of the individual candidate genes, remains to be experimentally validated, and the presence of conserved domains does not necessarily imply functional conservation. Therefore, further studies incorporating JA quantification, elucidation of transcriptional regulatory mechanisms, and functional analyses will be necessary to clarify the mechanism by which rhizobial inoculation promotes GL production.

## Supplementary Information

Below is the link to the electronic supplementary material.

Supplementary Material 1

Supplementary Material 2

Supplementary Material 3

Supplementary Material 4

Supplementary Material 5

Supplementary Material 6

Supplementary Material 7

Supplementary Material 8

Supplementary Material 9

## References

[1] Han Y, Pang X, Zhang X, Han R, Liang Z (2022) Resource sustainability and challenges: Status and competitiveness of international trade in licorice extracts under the Belt and Road Initiative. Global Ecol Conserv 34:e02014. 10.1016/j.gecco.2022.e02014

[2] Ozaki K, Shibano M, Kusano G, Watanabe H (2010) Aim for Production of *Glycyrrhiza Radix* in Japan (2) Selection of Pharmaceutically Fine Strains from *Glycyrrhiza uralensis* Fisher. Japanese Soc Pharmacogn 64:76–82

[3] Kusano G, Shibano M, Watanabe H, Ozaki K (2003) Pharmaceutical botanical studies on some *Glycyrrhiza* species. Yakugaku Zasshi 123:619–631. 10.1248/yakushi.123.619 12931658

[4] The Society of Japanese Pharmacopoeia (2021) The Japanese Pharmacopoeia Eighteenth Edition

[5] Chiyo N, Seki H, Kanamoto T, Ueda H, Kojoma M, Muranaka T (2024) Glycyrrhizin production in licorice hairy roots based on metabolic redirection of triterpenoid biosynthetic pathway by genome editing. Plant Cell Physiol 65:185–198. 10.1093/pcp/pcad161 PMC10873519 38153756

[6] Isah T (2019) Stress and defense responses in plant secondary metabolites production. Biol Res 52:39. 10.1186/s40659-019-0246-3 PMC6661828 31358053

[7] Davies MK, Jibran R, Zhou Y, Albert WN, Brummell AD, Jordan RB, Bowman LJ, Schwinn EK (2020) The evolution of flavonoid biosynthesis: a bryophyte perspective. Front Plant Sci 11:7. 10.3389/fpls.2020.00007 PMC7010833 32117358

[8] Vries S, Fürst-Jansen RMJ, Irisarri I, Ashok DA, Ischebeck T, Feussner K, Abreu NI, Petersen M, Feussner I, Vries J (2021) The evolution of the phenylpropanoid pathway entailed pronounced radiations and divergences of enzyme families. Plant J 107:975–1002. 10.1111/tps.15387 34165823

[9] Hawkins PJ, Oresnik IJ (2022) The *Rhizobium*-legume symbiosis: co-opting successful stress management. Front Plant Sci 12:796045. 10.3389/fpls.2021.796045 PMC8761673 35046982

[10] Kusaba I, Nakao T, Maita H, Sato S, Chijiiwa R, Yamada E, Arima S, Mareshige K, Ishimaru K, Akashi R, Suzuki A (2021) *Mesorhizobium* sp. J8 can establish symbiosis with *Glycyrrhiza uralensis*, increasing glycyrrhizin production. Plant Biotechnol 38:57–66. 10.5511/plantbiotechnology.20.1124a PMC8215473 34177325

[11] Yamamoto S, Shimomura A, Watanabe S, Kojoma M, Suzuki A (2025) Overwhelming glycyrrhizin production in Glycyrrhiza glabra induced by rhizobial symbiosis. J Nat Med 79:833–844. 10.1007/s11418-025-01918-2 PMC12228657 40415147

[12] Chen X, Hudson GA, Mineo C, Amer B, Baidoo EEK, Crowe SA, Liu Y, Keasling JD, Scheller HV (2023) Deciphering triterpenoid saponin biosynthesis by leveraging transcriptome response to methyl jasmonate elicitation in *Saponaria vaccaria*. Nat Commun 14:1–15. 10.1038/s41467-023-42877-0 PMC10625584 37925486

[13] Hayashi S, Reid DE, Lorenc MT, Stiller J, Edwards D, Gresshoff PM, Ferguson BJ (2012) Transient Nod factor-dependent gene expression in the nodulation-competent zone of soybean (*Glycine max* [L.] Merr.) roots. Plant Biotechnol J 10:995–1010. 10.1111/j.1467-7652.2012.00729.x 22863334

[14] Mochida K, Sakurai T, Seki H, Yoshida T, Takahagi K, Sawai S, Uchiyama H, Muranaka T, Saito K (2017) Draft genome assembly and annotation of *Glycyrrhiza uralensis*, a medicinal legume. Plant J 89:181–194. 10.1111/tpj.13385 27775193

[15] Amit R, Hirakawa H, Megha R, Shimizu Y, Shirasawa K, Kikuchi S, Seki H, Yamazaki M, Toyoda A, Isobe S, Muranaka T, Saito K (2022) Chromosome-scale genome assembly of Glycyrrhiza uralensis revealed metabolic gene cluster centred specialized metabolites biosynthesis. DNA Res 29:1–14. 10.1093/dnares/dsac043 PMC9763095 36535891

[16] Zhang Z, Li W, Meng F, Jin Y, Sun W, Kuang Q, Ren S, Liu W, Zhang L, Qin L, Lv B, Jia H, Li C (2026) Discover the maze-like network for glabridin biosynthesis. Nat Commun 17:1–14. 10.1038/s41467-026-68881-8 PMC12963581 41620399

[17] Broughton WJ, Dilworth MJ (1971) Control of leghemoglobin synthesis in snake beans. Biochem J 125:1075–1080. 10.1042/bj1251075 PMC1178271 5144223

[18] Prieto C, Barrios D (2020) RaNA-Seq: interactive RNA-Seq analysis from FASTQ files to functional analysis. Bioinformatics 36:1955–1956. 10.1093/bioinformatics/btz854 31730197

[19] Katoh K, Standley DM (2013) MAFFT multiple sequence alignment software version 7: improvements in performance and usability. Mol Biol Evol 30:772–780. 10.1093/molbev/mst010 PMC3603318 23329690

[20] Wong TKF, Ly-Trong N, Ren H, Demotte P, Baños H, Roger AJ, Susko E, Bielow C, De Maio N, Goldman N, Hahn MW, Dos Reis M, Vinh LS, Huttley G, Lanfear R, Minh BQ (2026) IQ-TREE 3: phylogenomic inference software using complex evolutionary models. Mol Biol Evol 43:msag117. 10.1093/molbev/msag117 PMC13191116 42085559

[21] Waterhouse AM, Procter JB, Martin DMA, Clamp M, Barton GJ (2009) Jalview Version 2—a multiple sequence alignment editor and analysis workbench. Bioinformatics 25:1189–1191. 10.1093/bioinformatics/btp033 PMC2672624 19151095

[22] Wang J, Chitsaz F, Derbyshire MK, Gonzales NR, Gwadz M, Lu S, Marchler GH, Song JS, Thanki N, Yamashita RA, Yang M, Zhang D, Zheng C, Lanczycki CJ, Marchler-Bauer A (2023) The conserved domain database in 2023. Nucleic Acids Res 51:D384–D388. 10.1093/nar/gkac1096 PMC9825596 36477806

[23] Livak KJ, Schmittgen TD (2001) Analysis of relative gene expression data using real-time quantitative PCR and the 2 – ∆∆CT method. Methods 25:402–408. 10.1006/meth.2001.1262 11846609

[24] Kim J, Lee J, Kang JS, Shim H, Kang D, Lee SH, Choi JP, Kim HS, Kim MS, Kim YI, Lee Y, Ziyaev Z, Shin YK, Bhak J, Yang TJ (2025) Contributions of interspecific hybrids to genetic variability in *Glycyrrhiza uralensis* and *G. glabra*. Sci Rep 15:1–13. 10.1038/s41598-025-92115-4 PMC11906797 40082484

[25] Pauwels L, Barbero GF, Geerinck J, Tilleman S, Grunewald W, Perez AC, Chico JM, Bossche RV, Sewell J, Gil E, García-Casado G, Witters E, Inzé D, Long J, Jaeger G, Solano R, Goossens A (2010) NINJA connects the co-repressor TOPLESS to jasmonate signalling. Nature 464:788–791. 10.1038/nature08854 PMC2849182 20360743

[26] Yao H, Wang F, Bi Q, Liu H, Liu L, Xiao G, Zhu J, Shen H, Li H (2022) Combined analysis of pharmaceutical active ingredients and transcriptomes of *Glycyrrhiza uralensis* under PEG6000-induced drought stress revealed glycyrrhizic acid and flavonoids accumulation via JA-mediated signaling. Front Plant Sci 13:920172. 10.3389/fpls.2022.920172 PMC9234494 35769299

[27] Shimoda Y, Nishigaya Y, Yamaya-Ito H, Inagaki N, Umehara Y, Hirakawa H, Sato S, Yamazaki T, Hayashi M (2020) The rhizobial autotransporter determines the symbiotic nitrogen fixation activity of Lotus japonicus in a host-specific manner. Proc Natl Acad Sci U S A 117:1806–1815. 10.1073/pnas.1913349117 PMC6983412 31900357

[28] Mun T, Bachmann A, Gupta V, Stougaard J, Andersen SU (2016) Lotus Base: an integrated information portal for the model legume *Lotus japonicus*. Sci Rep 6:39447. 10.1038/srep39447 PMC5180183 28008948

